# The Postmedieval Latvian Oral Microbiome in the Context of Modern Dental Calculus and Modern Dental Plaque Microbial Profiles

**DOI:** 10.3390/genes12020309

**Published:** 2021-02-22

**Authors:** Alisa Kazarina, Elina Petersone-Gordina, Janis Kimsis, Jevgenija Kuzmicka, Pawel Zayakin, Žans Griškjans, Guntis Gerhards, Renate Ranka

**Affiliations:** 1 Latvian Biomedical Research and Study Centre, Ratsupites Str. 1, LV-1067 Riga, Latvia; alisa.kazarina@biomed.lu.lv (A.K.); janiskimsis@gmail.com (J.K.); kuzmicka13@gmail.com (J.K.); pawel@biomed.lu.lv (P.Z.); 2 Institute of Latvian History, University of Latvia, Kalpaka Bulvaris 4, LV-1050 Riga, Latvia; e.petersone-gordina@hotmail.co.uk (E.P.-G.); guntis.gerhards@inbox.lv (G.G.); 3 Institute of Stomatology, Riga Stradins University, Dzirciema Str. 20, LV-1007 Riga, Latvia; z.griskjans@gmail.com

**Keywords:** ancient DNA, dental calculus, dental plaque, oral microbiome, metagenomics

## Abstract

Recent advantages in paleomicrobiology have provided an opportunity to investigate the composition of ancient microbial ecologies. Here, using metagenome analysis, we investigated the microbial profiles of historic dental calculus retrieved from archaeological human remains from postmedieval Latvia dated 16–17th century AD and examined the associations of oral taxa and microbial diversity with specific characteristics. We evaluated the preservation of human oral microbiome patterns in historic samples and compared the microbial composition of historic dental calculus, modern human dental plaque, modern human dental calculus samples and burial soil microbiota. Overall, the results showed that the majority of microbial DNA in historic dental calculus originated from the oral microbiome with little impact of the burial environment. Good preservation of ancient DNA in historical dental calculus samples has provided reliable insight into the composition of the oral microbiome of postmedieval Latvian individuals. The relative stability of the classifiable oral microbiome composition was observed. Significant differences between the microbiome profiles of dental calculus and dental plaque samples were identified, suggesting microbial adaptation to a specific human body environment.

## 1. Introduction

The oral cavity serves as a gateway to the human body. It is the place where food preprocessing occurs prior to passing it to the stomach and intestinal tract, has direct contact with incoming air on its way to the trachea and lungs, and contains numerous habitats, including the teeth, gingival sulcus, tongue, cheeks, hard and soft palates, and tonsils, which are colonized by bacteria [1]. At present, the oral microbiome is the second most studied human microbiome subtype after the gut microbiome and is known to hold over 700 species of bacteria belonging mostly to the Firmicutes, Proteobacteria, Actinobacteria and Bacteroidetes phyla [2]. Our knowledge about oral microbial communities is wide but not complete: approximately 57% of the known bacterial species are named, 13% of the species have been cultivated but not named, and 30% of the species remain uncultivated [3]. The community of oral bacteria in humans is also known to host a range of pathogenic or potentially pathogenic microorganisms when the healthy microbiome balance is disrupted. Bacteria of the oral cavity are known to cause a range of oral infectious diseases, such as tooth decay (caries), gum and root diseases, alveolar osteitis and tonsillitis [1]. It is also believed that dental plaque bacterial patterns may be linked with the overall health conditions, lifestyle and dietary preferences of the host [4].

Recent advantages in paleomicrobiology allow us to investigate the evolution of oral microbial ecologies that can contribute to a medical understanding of modern health and nutrition [5]. Ancient dental calculus—calcified oral plaque biofilm—is one of the best archaeological materials to provide us with information related to the ancient human microbiome and its interaction with its host. Dental calculus represents over 600 different bacterial taxa that are common to saliva and dental plaque [1,6,7]. However, searching for human oral microbiome patterns within ancient dental calculus microbial profiles demands a careful interpretation of modern versus ancient microbiome differences. Modern oral microbiome research operates mostly with living bacterial biofilms because dental calculus is not as widespread as it was historically. At the same time, specific differences in bacterial patterns have been discovered between dental plaque and dental calculus, which can be explained by plaque maturation processes [8].

Overall, the application of metagenomic technologies has provided a new tool to directly access the genetic information of a microbial community in historical samples, allowing us to address a number of questions, such as the exact nature of the bacteria present in particular historical periods and geographical regions.

Here, using metagenome analysis, we aimed to investigate the microbial profiles of historic dental calculus retrieved from archaeological human remains from postmedieval Latvia dated 16–17th century AD. We evaluated the preservation of human oral microbiome patterns in historic samples by comparison to modern human dental plaque and calculus samples and burial soil microbiota and concluded that the majority of microbial DNA in historic calculus originates from the oral microbiome with little impact of the burial environment. We also tested the associations of oral taxa and microbial diversity in historic dental calculus with specific characteristics.

## 2. Materials and Methods 

### 2.1. Archaeological Sample Characteristics, Collection and Burial Site Data

During this study, 15 historic human dental calculus samples were examined, representing 6 Latvian cemeteries in 4 cities: the capital Riga (Dom Square cemetery, 56.94902, 24.10473; St. Peter’s Church cemetery, 56.94752, 24.10928; St. Gertrude’s Church cemetery, 56.95799, 24.12172), Cesis (St. John’s Church cemetery, 57.31213, 25.27168), Kuldiga (Church of the Holy Trinity, Roman Catholic Church, 56.96765, 21.96943) and Jelgava (St. Trinity’s Church, 56.65239, 23.72897). All samples were dated to 16–17th century AD (Table 1).

Most individuals were in their young adulthood to middle age (20–60 years of age) at the time of death. Sample ZA_7C was suspected to represent a teenager (14–15 years of age). Prior to dental calculus sample collection, archaeological skeletons were inspected for the presence of any disease-specific leisure signs. Mouth cavities were inspected for the presence of oral disease lesions [9]. Four archaeological skeletons were found to exhibit tooth decay signs, 10 skeletons exhibited specific and nonspecific bone lesions, and for four skeletons, no signs of diseases were observable (Table 1). The teeth and the alveolar bone appeared to be macroscopically sound without traces of periodontal disease. To determine the burial period of the samples, the stratigraphy method was used together with the evaluation of archaeological finds [10,11]. The approximate age at death was determined by evaluating degenerative changes in the pubic symphysis and using the auricular surface standard method [12,13].

Random soil samples were collected together with bone samples during the excavation process to evaluate the microbiome composition of the burial environment. Burial soil samples were collected from two cemeteries in Riga, Latvia: Dome Square cemetery (samples Z5 and Z6_sk) and St. Peter’s Church cemetery (samples Z7 and Z7_sk). Soil samples were collected at the burial depth from the middle section of the skeleton, i.e., between the ribcage and the pelvis, approximately 5–10 cm above the bones. All samples were packed in plastic bags and stored separately from the bone samples under the same conditions to avoid any further contamination.

### 2.2. Modern Dental Calculus and Modern Dental Plaque Sample Collection

Modern dental supragingival calculus samples (*n* = 4) were collected at the Institute of Stomatology (Riga Stradins University, Riga, Latvia) by a professional dentist during the process of routine dental cleaning (Table 2). Dental calculus samples were collected with the use of a dental scaler. The exact location of the sample taken was selected arbitrarily by the dentist. Modern dental supragingival plaque samples (*n* = 20) were collected by rubbing a cotton swab over the supragingival sites of the teeth. All samples were collected with patient consent, and the study was reviewed and approved by Riga Stradins University Research Ethics Committee, decision no. 6-3/4/5 (25 April 2019).

### 2.3. DNA Isolation

Ancient DNA (aDNA) handling in laboratory conditions requires special care and precautions to eliminate all possible contamination from modern DNA sources. This study strictly followed specific guidelines developed exclusively for aDNA research [14,15]. aDNA handling protocols consist of actions prescribing facility preparation prior to aDNA handling, instrument treatment, facility worker equipment and archaeological material processing. Ancient DNA facility preparation required regular ultraviolet (UV) light irradiation and weekly surface and floor cleaning actions with 5% sodium hypochloride (NaClO) solution. All instruments involved in aDNA procedures were treated similarly by washing with 5% NaOCl solution and irradiating with UV light before and after each procedure. Archaeological samples were processed one at a time.

The aDNA facility consisted of three strictly separated chambers, each serving its defined purpose. Archaeological material preprocessing and pulverized sample incubation occurred in one facility chamber, and another two chambers were devoted to aDNA isolation and library preparation. The fourth chamber (buffer zone) separated the aDNA facility from the rest of the laboratory.

Archaeological tooth samples carrying the desired dental calculus remains were first immersed in 5% NaOCl solution, rinsed with nuclease-free water and irradiated for 2 h with UV light (6 J/cm2 at 254 nm). Samples were then left to dry overnight at room temperature. The next day, dental calculus was cautiously removed from the surface of the teeth with a scalpel and was ground inside a tube with a sterile microbiological stick. Laboratory blank samples (BC and BC_sk) were processed simultaneously with archaeological samples. DNA extraction was performed as described previously [16,17].

Burial soil and modern dental calculus samples were processed in another facility to avoid possible cross-contamination with aDNA samples. Burial soil samples that were chosen to represent the soil microbiome of the burial environment of St. Peter’s Church cemetery (samples Z5 and Z6_sk) and St. Gertrude’s Church cemetery (samples Z7 and Z7_sk) underwent the same procedures of DNA extraction and purification as the aDNA samples. Modern dental calculus samples were washed with 5% NaOCl solution and were then rinsed with nuclease-free water. Furthermore, together with the modern dental plaque samples, modern dental calculus samples underwent a DNA extraction process.

All resultant DNA samples were inspected with a Qubit dsDNA HS Assay Kit (Life Technologies, Carlsbad, CA, USA) to estimate the resultant DNA concentration (Table S1, Supplementary Materials).

### 2.4. Library Preparation and Shotgun Metagenomics Sequencing

Before library preparation, modern dental calculus, modern dental plaque DNA samples, and burial soil DNA samples Z6_sk (St. Peter’s Church cemetery) and Z7_sk (St. Gertrude’s Church cemetery), underwent an additional DNA fragmentation step using the Ion Shear^TM^ Plus Reagent Kit (Ion Torrent™, Thermo Fisher Scientific, Waltham, MA, USA) following the manufacturer’s instructions. The fragmentation conditions were selected according to the desired fragment size (150–250 bp).

For the historic dental calculus samples and for burial soil samples Z5 (St. Peter’s Church cemetery) and Z7 (St. Gertrude’s Church cemetery), the DNA fragmentation step was omitted to ensure the capture of short DNA fragments that are believed to represent aDNA [18,19,20]. DNA samples underwent a size-selection procedure to remove DNA fragments larger than 250 bp using NucleoMag® NGS Clean-up and Size Select magnetic beads (Macherey-Nagel), and for library preparation, an Ion Plus Fragment Library Kit (Ion Torrent™) was used. To evaluate sample contamination from laboratory sources, two laboratory control samples were processed with the historical DNA samples and sequenced: aDNA extraction blank sample (BC) and aDNA extraction blank sample, which also underwent a DNA fragmentation step (BC_sk).

Preparation of all sequencing libraries followed the same steps regardless of sample origin. Specific barcodes were ligated, and libraries underwent amplification and quality assessment using an Agilent High Sensitivity DNA Kit and Bioanalyzer 2100 (Agilent Technologies, Santa Clara, CA, USA) following the manufacturer’s protocol. Sequencing was performed on an Ion ProtonTM System (Ion Torrent™, Thermo Fisher Scientific, Waltham, MA, USA).

All raw historic calculus DNA sequencing data are publicly available at the ENA under accession PRJEB40382.

### 2.5. Sequencing Data Analysis

Sequencing data preprocessing on the local Ion Torrent Proton server included initial quality control steps as well as data assignment to each individual sample. Barcodes and sequencing adapters, together with polyclonal and low-quality sequences, were filtered by Proton software during the first post-sequencing data handling step. The resultant data were exported for further manipulations in the form of BAM files. The resulting exported BAM files were initially quality-processed using the Galaxy public server [21,22]. Briefly, overrepresented sequences were removed, and reads with quality PHRED scores <20 were excluded. Sequencing data taxonomic assignment was performed with Kraken2 v2.0.7 using the standard Kraken2 database [23]. Bracken (Bayesian Reestimation of Abundance with Kraken) was used to compute the abundance of species in DNA sequences from a metagenomics sample [24].

Pavian R application [25] was used to further manipulate Kraken/Bracken taxonomy report files, generate quality assignment and prepare data for statistical analysis and representation, which was done using the MicrobiomeAnalyst (https://www.microbiomeanalyst.ca/ (accessed on 23 December 2020)) web application [26,27].

The Bayesian analysis-based program SourceTracker [28] was used to evaluate the possible source of predominant microbial signatures of historic dental calculus samples to enable oral microbiome preservation assessment and to track the influence of exogenous microbial contamination. All source files, which were included in the pipeline, were defined from these study samples. There were five sources defined: modern supragingival calculus, modern supragingival plaque, two aDNA extraction blanks and burial soil. The open-source R package decontam (https://github.com/benjjneb/decontam (accessed on 23 December 2020)) [29] was used for the identification and removal of laboratory contaminants in metagenomics data. Low-abundance species were removed by applying a hard cutoff (0.001% abundance).

The authenticity of historic specimen microbiome data was confirmed using DNA damage patterns. This method is based on the hypothesis that DNA deamination rates increase over time [19], and characteristic features of damaged DNA patterns may confirm the origin of the DNA. For this purpose, read files of historic dental calculus samples were analyzed using MALT 0.5.0 [30] (https://software-ab.informatik.uni-tuebingen.de/download/malt/welcome.html (accessed on 23 December 2020)), using all complete bacterial genomes available from NCBI Assembly in August 2020 as a reference. Bacterial reads were extracted using SAMtools, mapped to the reference genome of the prevalent oral microorganism *Olsenella* sp. oral taxon 807 and *Actinomyces* sp. oral taxon 414, and DNA deamination rates were calculated using mapDamage [31] by the EAGER pipeline [32].

### 2.6. Statistical Analysis

Intergroup differences in alpha diversity were assessed by the Shannon diversity index. Beta diversity was tested by permutational multivariate analysis of variance (PERMANOVA), a nonparametric multivariate statistical test [33] presented by principal coordinates analysis (PCoA). Hierarchical clustering was visualized by dendrogram and heatmap analysis using the Bray–Curtis similarity index and Ward clustering algorithm.

Intergroup differences at the species level were analyzed using the Kruskal–Wallis test and the linear discriminant analysis (LDA) effect size (LEfSe) method [34] with default settings on the MicrobiomeAnalyst website; the threshold on the logarithmic LDA score for discriminative features was set to 2.0, and the false discovery rate (FDR)-adjusted *p* value cutoff was set to 0.05.

## 3. Results

### 3.1. Sequencing Data and Ancient DNA Authentication

In total, a set of 43 DNA samples was analyzed: historic supragingival calculus (15 samples), modern supragingival calculus (4 samples), modern supragingival plaque (20 samples) and burial soil (4 samples). A total of 0.11 billion sequences were generated, with an average of 2.6 million reads (standard deviation (SD) = 1.3 million reads) per sample (Table S1, Supplementary Materials). Among them, 19.34% of the reads were classified to the bacterial species level, with an average of 0.51 million reads (65248–1928089; SD 0.40 million reads) per sample.

For both blank samples, despite the sufficient sequencing depth, much lower amounts of classified reads were obtained: only 2260 (1.27%) and 688 (1.29%) reads were classified to the bacterial species level for the aDNA extraction blank samples BC and BC_sk, respectively. A large proportion of the obtained reads in blank samples seem to be sequencing artifacts that could not be classified. In total, in the aDNA extraction blanks, 14 bacterial species were detected; the vast majority of the reads (87.35%) belonged to the *Delftia* genus (Figure S2A, Supplementary Materials). In other studies, low bacterial diversity was routinely obtained from laboratory extraction controls [35], while the *Delftia* genus has been detected in sequenced negative ‘blank’ controls and detected as a contaminant of amplification kits [36].

SourceTracker analysis demonstrated that the historical dental calculus sample group in this study had a predominant dental calculus microbial signature, indicating sufficient preservation of the oral microbiome in a mineralized oral plaque biofilm (Figure S1, Supplementary Materials). A modern dental plaque signature was also present, but in lower amounts. Traces of exogenous contamination defined from the burial soil source and laboratory blanks were low, indicating a low impact of laboratory and environmental contamination on the study samples.

For the authentication of oral microbiome preservation in historic dental calculus samples, the DNA damage pattern of two of the most prevalent oral microorganisms, *Olsenella* sp. oral taxon 807 and *Actinomyces* sp. oral taxon 414 was evaluated. The analysis revealed signs of cytosine to thymine substitutions at the ends of DNA fragments, characteristic of aDNA (Figures S3 and S4, Supplementary Materials).

### 3.2. Taxonomical Analysis of Microbial Profiles at the Species Level

**Figure 1:**
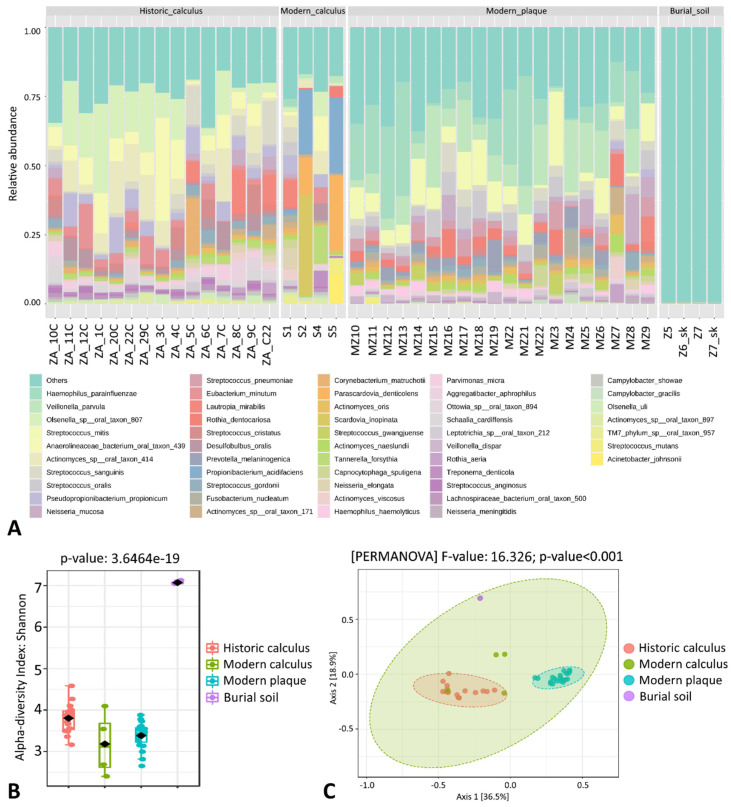
Species-level comparison of bacterial profiles in 16–17th century human archaeological dental calculus, modern dental calculus, modern dental plaque and burial soil samples: (**A**) Stacked plots of the taxonomic classification. The abundances of the most abundant species are shown; (**B**) Shannon diversity analysis; (**C**) Principal coordinates analysis (PCoA) derived from Bray–Curtis distances among samples of the four groups (*p* < 0.001 by PERMANOVA). For each axis, in square brackets, the percent of variation explained was reported.

Based on decontam analysis and low prevalence (prevalence filter: 0.001%), a total of 1842 OTUs were removed, and 2223 features remained after data filtering within all 43 samples. During the metagenomics analysis of the sequencing data, differences in the overall microbial patterns at the species level were observed for the four sample groups (Figure 1A).

The 10 most abundant bacterial species within the group of modern dental plaque samples were *Veillonella parvula* (14.92%), *Haemophilus parainfluenzae* (12.31%), *Streptococcus mitis* (7.10%), *Streptococcus oralis* (4.37%), *Neisseria mucosa* (4.23%), *Prevotella melaninogenica* (3.46%), *Streptococcus pneumoniae* (2.82%), *Streptococcus sanguinis* (2.56%), *Fusobacterium nucleatum* (2.53%), and *Rothia dentocariosa* (1.97%) (Table S2, Supplementary Materials). In contrast, the 10 most abundant bacterial species within the modern calculus samples were *Propionibacterium acidifaciens* (13.33%), *Parascardovia denticolens* (10.25%), *Scardovia inopinata* (8.57%), *Lautropia mirabilis* (3.66%), *Actinomyces* sp. oral taxon 414 (3.32%), *Pseudopropionibacterium propionicum* (3.06%), *Acinetobacter johnsonii* (2.98%), *Neisseria elongata* (2.94%), *Olsenella* sp. oral taxon 807 (2.03%), and *Streptococcus sanguinis* (2.01%).

Historic dental calculus samples represented a slightly different pattern of the most abundant species, the first 10 of which were *Olsenella* sp. oral taxon 807 (11.83%), *Actinomyces* sp. oral taxon 414 (8.29%), *Anaerolineaceae* bacterium oral taxon 439 (7.84%), *Pseudopropionibacterium propionicum* (5.20%), *Streptococcus sanguinis* (4.96%), *Eubacterium minutum* (3.53%), *Desulfobulbus oralis* (3.51%), *Lautropia mirabilis* (3,42%), *Streptococcus cristatus* (2.85%), and *Ottowia* sp. oral taxon 894 (1.82%) (Table S2, Supplementary Materials).

Additionally, the presence of several bacteria that were among the most abundant species in the archaeological calculus samples was detected in the blank samples but in much lower proportions (Figure S2A, Supplementary Materials). As the blank samples were processed simultaneously with the historical dental calculus samples, these results indicate that the contamination was most likely passed from the archaeological samples to the blank samples.

Burial soil samples showed much higher microbial alpha diversity than the oral microbiome samples (ANOVA, *F* value = 111.31, *p* < 0.001) (Figure 1B); the ten most abundant species in soil samples were *Sorangium cellulosum* (1.11%), *Polaromonas* sp. JS666 (0.83%), *Luteitalea pratensis* (0.69%), *Gemmatirosa kalamazoonesis* (0.52%), *Rhodopseudomonas palustris* (0.48%), *Streptomyces venezuelae* (0.40%), *Planctomycetes* bacterium ETA A1 (0.39%), *Streptomyces lydicus* (0.39%), *Achromobacter xylosoxidans* (0.39%), and *Streptomyces hygroscopicus* (0.35%), while other species composed 88.28% of the sequencing reads (Figure S2B, Supplementary Materials). The most abundant soil-related bacterial species were also detected in historical dental calculus samples but in much smaller amounts (Table S3, Supplementary Materials).

**Figure 2:**
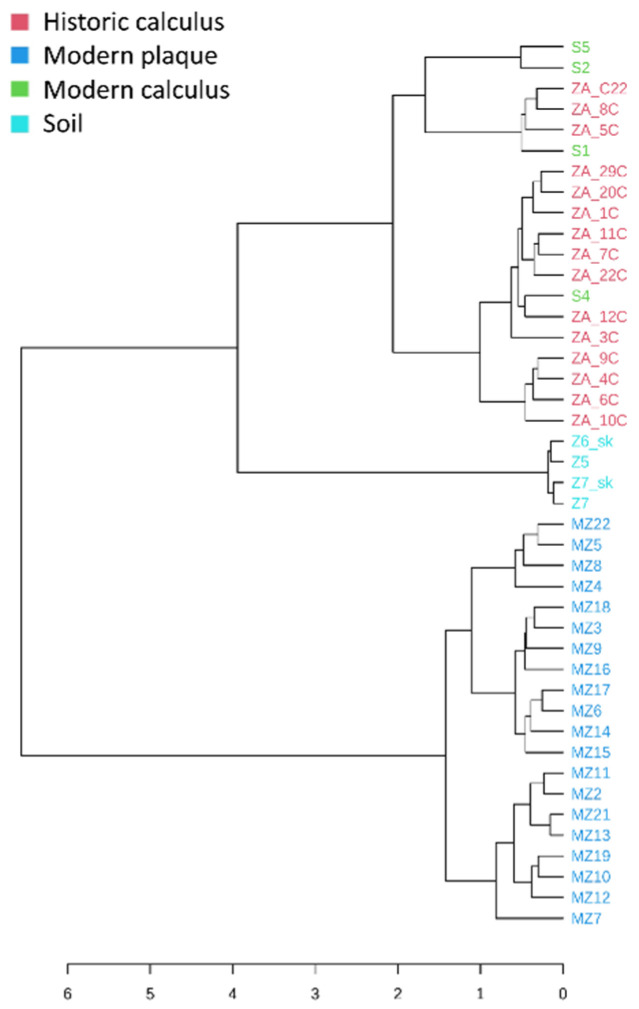
Species-level clustering dendrogram based on the Bray–Curtis index and Ward clustering algorithm. Samples with more similar species profiles were clustered closer together.

Microbial beta diversity analysis found significant separation between burial soil, historic dental calculus and modern dental plaque samples (PERMANOVA, *F* value = 16.326, *p* < 0.001) (Figure 1C). Modern calculus samples were located closer to the historic calculus sample cluster, and dental plaque samples and burial soil samples formed tightly separated aggregates. A dendrogram analysis using the Bray–Curtis Index and the Ward clustering method also showed clear separation of the dental plaque, burial soil and dental calculus samples regardless of the origin, based on the normalized relative abundance of identified bacterial species (Figure 2).

**Figure 3:**
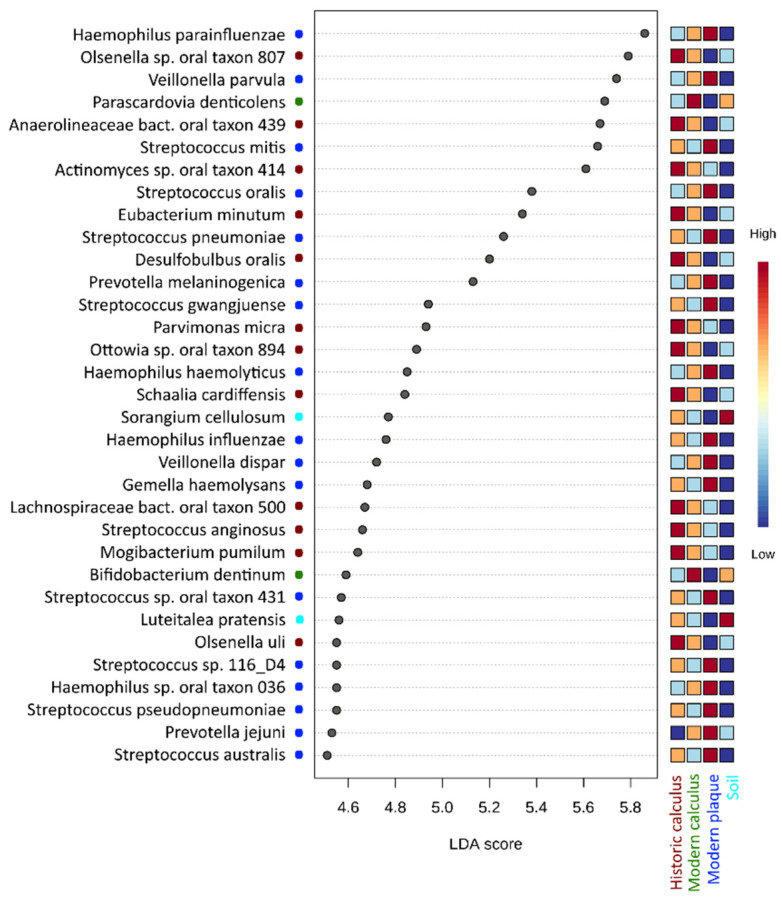
Linear discriminant analysis (LDA) combined with effect size measurements (LEfSe) identified species that enabled discrimination between the microbiotas of ancient calculus, modern calculus, modern plaque and burial soil samples. False Discovery Rate (FDR)-adjusted *p* value cutoff: 0.05; logarithmic LDA score ≥ 4.5.

LEfSe analysis identified 34 differentially abundant bacterial taxa in the microbiotas of historic dental calculus, modern calculus, modern dental plaque and burial soil samples (LDA score [log 10] > 4.5) (Figure 3). Within the most significant LEfSe results (LDA score [log10] > 5.0) at the species level, we found six microbial species that were mainly attributed to the modern dental plaque sample group (*Haemophilus parainfluenzae*, *Veillonella parvula*, *Streptococcus mitis*, *Streptococcus oralis, Streptococcus pneumoniae, Prevotella melaninogenica*) and five species were attributed to the historic dental calculus sample group (*Olsenella* sp. oral taxon 807, *Anaerolineaceae* bacterium oral taxon 439, *Actinomyces* sp. oral taxon 414, *Eubacterium minutum* and *Desulfobulbus oralis*) (Figure 3).

### 3.3. Taxonomic Analysis of Microbial Profiles of Historic Dental Calculus Samples at the Species Level

Furthermore, a detailed metagenomic analysis of the microbiome signature of the historical dental calculus samples was performed. As shown in Figure 1A, the number of sequences aligned to particular oral bacteria differed among the subjects analyzed. Sample ZA_5C had a very high level of *Corynebacterium matruchotii* (20.31%), ZA_1C—*Olsenella* sp. oral taxon 807 (33.73%), and ZA_3C—*Anaerolineaceae* bacterium oral taxon 439 (40.12%).

The taxonomic composition of the historic dental calculus samples was studied at the species level based on the sample characteristics, including sex, age group (≤40 years old and >40 years old), possible lifestyle (town and countryside), tooth type and presence/absence of caries. No significant differences in alpha and beta diversity were observed (Figures S5 and S6, Supplementary Materials), and no significant features were detected during LEfSe analysis either.

No oral pathogen species were significantly associated with the prevalence of caries by univariate analysis (Kruskal–Wallis test, false discovery rate corrected *p* value greater than 0.05), including *Streptococcus mutans* and members of the periodontitis-associated ‘red-complex’ (*Treponema denticola*, *Tannerella forsythia*, *Porphyromonas gingivalis*) [37]. 

## 4. Discussion

In our study, historic dental calculus, modern dental calculus and oral plaque samples were analyzed by a shotgun metagenomics approach. For the first time, oral microbiome patterns of postmedieval Latvian individuals were studied, and the results of this study showed that despite some burial environmental contamination, historic dental calculus samples provide a reliable snapshot of bacterial oral communities from past individuals.

The path of historical bacterial research has various challenges. The implementation of multiple methodologies (from laboratory practices to data analysis quality control pipelines) helps to improve validation of aDNA data in ancient dental calculus samples; however, environmental contamination (from both burial soil and laboratory environments) in samples is a reality. Extraction blank controls should always be included and sequenced together with archaeological samples to monitor in-lab contamination, and caution must be taken in interpreting the data [18]. Moreover, as we observed in our study for the blank controls, low-level contamination can also happen from one sample to another during the workflow, thus especial care should be taken when samples are processed in batches. In our study, we avoided this probable issue by processing the archaeological samples one at a time; however, this approach is time-consuming and could not be feasible on a larger scale.

The analysis of burial soil samples together with archaeological samples was suggested to assess the environmental contamination [38]. In our study, a clear separation of burial soil microbiomes from both ancient and modern oral microbiomes was observed. Several soil bacterial species were detected in ancient dental calculus microbiomes but were nearly absent in modern oral samples, although the ancient and modern calculus samples were clustered tightly together. This finding can be easily explained by the direct impact of the burial environment. However, industrialization, urbanization, and modern food processing have dramatically reduced human contact with soil microorganisms. Traces of dirt may be incorporated into dental calculus over a lifetime of eating food that is not fully cleaned. This problem is further complicated by the fact that some microorganisms tend to colonize multiple environmental niches, including soil and oral cavity. For example, an environmentally ubiquitous opportunistic pathogen *Pseudomonas aeruginosa* is known to be involved in multiple oral infections [39,40]. Additionally, a recent study proposed a novel environmental microbiome hypothesis, stressing a close linkage between the human intestinal microbiome and the soil microbiome, which has evolved during evolution [41]. This theory might also affect the ancient human oral microbiome. However, in our study, we were not able to demonstrate the ancient origin of the soil taxa found in historic calculus samples due to the relatively low sequencing coverage. Additional studies are required that would shed light on this question.

The vast majority of bacterial species detected in historical dental calculus belonged to the oral microflora, and despite some individual variations, the microbiome composition did not differ significantly from that of the modern samples. The similarities between postmedieval and modern calculus samples can be explained by the relatively short time span and the same geolocation. Additionally, the majority of archaeological samples in our study were from individuals who possibly had better access to foods that were linked with higher social status, such as soft dietary carbohydrates (finely ground bread, sweets) and meat [42]. On the other hand, we did not identify significant differences between ancient samples that were grouped based on sex, age, and lifestyle. This result is consistent with previous studies [8,38,43] suggesting the relative stability in the composition of the oral microbiome at definite time points.

The most abundant bacterial taxa detected in our historical calculus samples included several commensal bacterial species that are commonly found in the human oral cavity, such as *Streptococcus sanguinis*, *Streptococcus cristatus* and *Lautropia mirabilis*. The periodontal pathobiont *Desulfobulbus oralis* was present among the 10 most abundant bacterial species from postmedieval dental calculus samples, which is known for its ability to trigger a proinflammatory response in the oral epithelium [44]. Additionally, many oral pathogens that are involved in the etiology of caries, such as *Streptococcus mutans*, or periodontal disease, including three Gram-negative species known as the ‘red-complex’—*Porphyromonas gingivalis*, *Tannerella forsythia*, and *Treponema denticola*—were detected in our historical calculus samples. While archaeological samples included in our study were without clear evidence of periodontal disease, the presence of periodontal organisms is not surprising given the complex etiology of periodontal disease and the fact that studies of periodontitis in ancient populations pose some technical challenges [45]. Future studies with larger sample sizes, including both periodontal-positive and periodontal-negative individuals, are needed to determine the microbial association with the disease in postmedieval Latvian individuals.

The analysis of taxonomic results showed that dental calculus samples tended to cluster together, and dental plaque samples formed a separate cluster. In a recent study, it was suggested that a microbial profile gap appears between dental plaque and dental calculus due to the processes of microbial biofilm maturation [8]. Here, when comparing postmedieval dental calculus samples to modern dental plaque and calculus samples within the same geographical location, the results indicate that biofilm type can have a greater impact on microbial communities than chronological origin of the sample (historic vs. modern). Although we can locate potentially pathogenic bacteria on the map of postmedieval dental calculus from Latvia, additional studies are required to reveal paths of microbial coexistence and disease-specific microbial profiles in dental calculus ecosystems.

There are several limitations in our study. First, the limited historical calculus sample size could have prevented the detection of the differences in microbiome composition. Tooth type is known to be a factor influencing plaque/calculus microbial communities [46,47]; however, we were not able to control for the tooth type used in our study. Additionally, the possible lifestyles of the individuals was determined based on the burial place and historical evidence of burial practices in postmedieval Latvia and thus does not include all possibilities of lifestyle/dietary/health changes during the lifetime.

## 5. Conclusions

Overall, the results showed good preservation of ancient DNA in historical dental calculus samples, providing reliable insight into the composition of the postmedieval oral microbiome of Latvian individuals, and the relative stability of the classifiable oral microbiome composition was observed. Significant differences between microbiome profiles of dental calculus and dental plaque samples were identified, suggesting microbial adaptation to a specific human body environment.

## Figures and Tables

**Table 1:** Characteristics of archaeological samples.

Sample ID	City	Cemetery	Century, AD	Year of Excavation	Age (Years)	Sex	Lifestyle	Possible Diseases	Tooth Type
ZA_1C	Riga	Dom Square	16–17th	1986	30–40	Male	Town/city, middle classes	Inflammation of the clavicle	Molar
ZA_3C	Riga	Dom Square	16–17th	1986	45–50	Female	Town/city, middle classes	Caries	Incisor
ZA_4C	Riga	St. Peter’s Church	16–17th	2004	30–35	Male	Town/city	-	Incisor
ZA_5C	Riga	St. Peter’s Church	16–17th	2004	25–30	Male	Town/city	Fractured rib and left arm’s fracture	Incisor
ZA_6C	Riga	St. Peter’s Church	16–17th	2004	20–25	Male	Town/city	Non-specific inflammation of the lower leg bone surface	Premolar
ZA_7C	Riga	St. Gertrude’s Church	16–17th	2006	14–15	Unknown	Countryside, commuter town	-	Incisor
ZA_8C	Riga	St. Gertrude’s Church	17th	2006	45–50	Male	Countryside, commuter town	Deforming arthrosis (joints)	Incisor
ZA_9C	Riga	St. Gertrude’s Church	16–17th	2006	25–30	Male	Countryside, commuter town	Vertebral fracture with local inflammation	Molar
ZA_10C	Riga	St. Gertrude’s Church	16–17th	2006	55–60	Male	Countryside, commuter town	Caries, tooth root abscess, arthritis in joints	Molar
ZA_11C	Cesis	St. John’s Church	17th	2015	35–40	Male	Town/city, aristocracy	Multiple tooth decay, toe osteomyelitis	Incisor
ZA_12C	Cesis	St. John’s Church	17th	2015	35–40	Female	Town/city, aristocracy	Non-specific inflammatory process on the surface of the leg bones	Premolar
ZA_20C	Kuldiga	Church of the Holy Trinity, Roman Catholic Church	16–17th	2015	45–50	Female	Town/city, lower classes	-	Molar
ZA_22C	Kuldiga	Church of the Holy Trinity, Roman Catholic Church	16–17th	2015	30–35	Female	Town/city, lower classes	-	Incisor
ZA_29C	Jelgava	St. Trinity’s Church	16–17th	2009	35–40	Female	Town/city, aristocracy	Caries, arthritis in joints, non-specific inflammation	Premolar
ZA_C22	Riga	St. Gertrude’s Church	17th	2006	40–50	Unknown	Countryside, commuter town	Deforming arthrosis, pelvic joint	Incisor

**Table 2:** Characteristics of modern samples.

	Sample Type	Sex	Teeth Health *	Age (Years) **
MZ2	Supragingival dental plaque	Male	healthy	23
MZ3	Supragingival dental plaque	Male	treated	23
MZ4	Supragingival dental plaque	Male	treated	28
MZ5	Supragingival dental plaque	Female	treated	23
MZ6	Supragingival dental plaque	Male	treated	24
MZ7	Supragingival dental plaque	Female	treated	24
MZ8	Supragingival dental plaque	Male	treated	24
MZ9	Supragingival dental plaque	Male	treated	22
MZ10	Supragingival dental plaque	Female	healthy	23
MZ11	Supragingival dental plaque	Female	treated	22
MZ12	Supragingival dental plaque	Female	treated	22
MZ13	Supragingival dental plaque	Female	healthy	22
MZ14	Supragingival dental plaque	Female	treated	23
MZ15	Supragingival dental plaque	Female	treated	23
MZ16	Supragingival dental plaque	Female	treated	22
MZ17	Supragingival dental plaque	Female	treated	22
MZ18	Supragingival dental plaque	Female	treated	19
MZ19	Supragingival dental plaque	Male	treated	22
MZ21	Supragingival dental plaque	Female	healthy	23
MZ22	Supragingival dental plaque	Female	healthy	20
S1	Supragingival dental calculus	Male	treated	N/A
S2	Supragingival dental calculus	Male	treated	N/A
S4	Supragingival dental calculus	Female	treated	N/A
S5	Supragingival dental calculus	Male	treated	N/A

* treated: dental fillings were present. ** N/A—not available.

## Data Availability

All raw historic calculus DNA sequencing data are publicly available at the ENA under accession PRJEB40382.

## Supplementary Materials

The following are available online at https://www.mdpi.com/2073-4425/12/2/309/s1, Figure S1: Application of SourceTracker methodology to identify the percentage contribution of each potential source to the microbiome of historic dental calculus samples using a Bayesian model. Figure S2: Analysis of bacterial profiles in blank and burial soil samples: (**a**) Species-level composition of laboratory blank samples; (**b**) Species-level composition of burial soil samples. The most abundant 30 species are shown. Figure S3: Damage plots generated from sequencing reads obtained from 16th–17th century human archaeological dental calculus samples demonstrated a pattern characteristic of ancient DNA. Reads were mapped to the reference genome of *Actinomyces* sp. oral taxon 414. Representative plots from three historic calculus samples of the C > T and G > A nucleotide transition frequencies at the 5′ and 3′ ends of DNA fragments, respectively, are shown. Figure S4: Damage plots generated from sequencing reads obtained from 16th–17th century human archaeological dental calculus samples demonstrated a pattern characteristic of ancient DNA. Reads were mapped to the reference genome of *Olsenella* sp. oral taxon 807. Representative plots from three historic calculus samples of the C > T and G > A nucleotide transition frequencies at the 5′ and 3′ ends of DNA fragments, respectively, are shown. Figure S5: Shannon diversity analysis and permutational multivariate analysis of variance of bacterial profiles in 16th–17th century human archaeological dental calculus based on three different variables: sex, possible lifestyle and age group. *p* values are shown. Figure S6: Shannon diversity analysis and permutational multivariate analysis of variance of bacterial profiles in 16th–17th century human archaeological dental calculus based on two different variables: tooth type and presence/absence of caries. *p* values are shown. Table S1: Sequenced sample data. Table S2: The relative abundance of bacterial species in ancient calculus, modern calculus and modern plaque and burial soil samples. The 100 most abundant species are shown. Table S3: The relative abundance of bacterial species in burial soil, ancient calculus, modern calculus, and modern plaque samples. The 100 most abundant burial soil species are shown.
